# Supplementary material for: Development of a Sericin Hydrogel to Deliver Anthocyanins from Purple Waxy Corn Cob (Zea mays L.) Extract and In Vitro Evaluation of Anti-Inflammatory Effects
Source: Pharmaceutics. 2022 Mar 6;14(3):577. doi: 10.3390/pharmaceutics14030577 (PMC8951468; doi:10.3390/pharmaceutics14030577)
Supplement: Supplementary file 1 [file pharmaceutics-14-00577-s001.zip › pharmaceutics-1567478-supplementary.pdf]

Supplementary Materials: Development of a Sericin Hydrogel to Deliver Anthocyanins from Purple Waxy Corn Cob (*Zea mays* L.) Extract and In Vitro Evaluation of Anti-Inflammatory Effects

Nattawadee Kanpipit, Natsajee Nualkaew, Worawikunya Kiatponglarp, Aroonsri Priprem and Suthasinee Thapphasaraphong

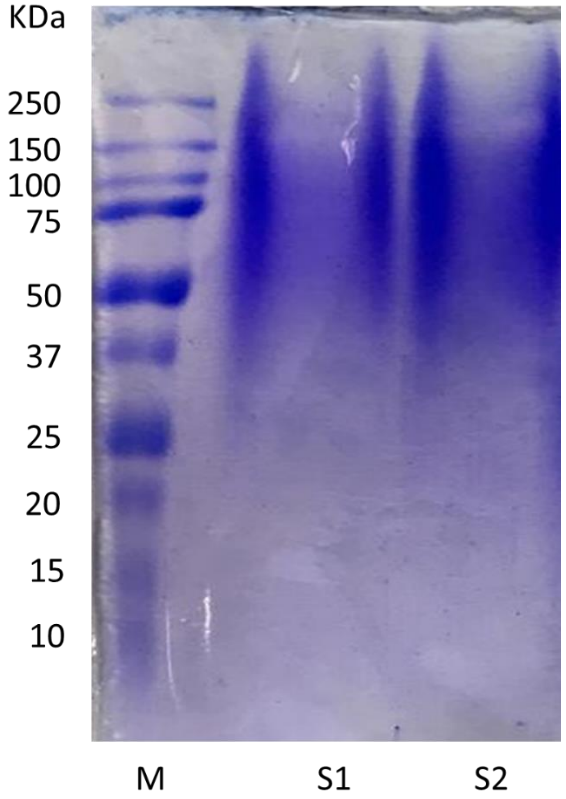

Figure S1. SDS-PAGE analysis of sericin extracted from silkworm cocoons (J108) by a High Temperature and High Pressure Degumming.

M = Protein marker,

S1 = Sericin extracted from silkworm cocoons (J108) by a High Temperature and High Pressure Degumming before storage condition (4 °C, 1 year)

S2 = Sericin extracted from silkworm cocoons (J108) by a High Temperature and High Pressure Degumming after storage condition (4 °C, 1 year)

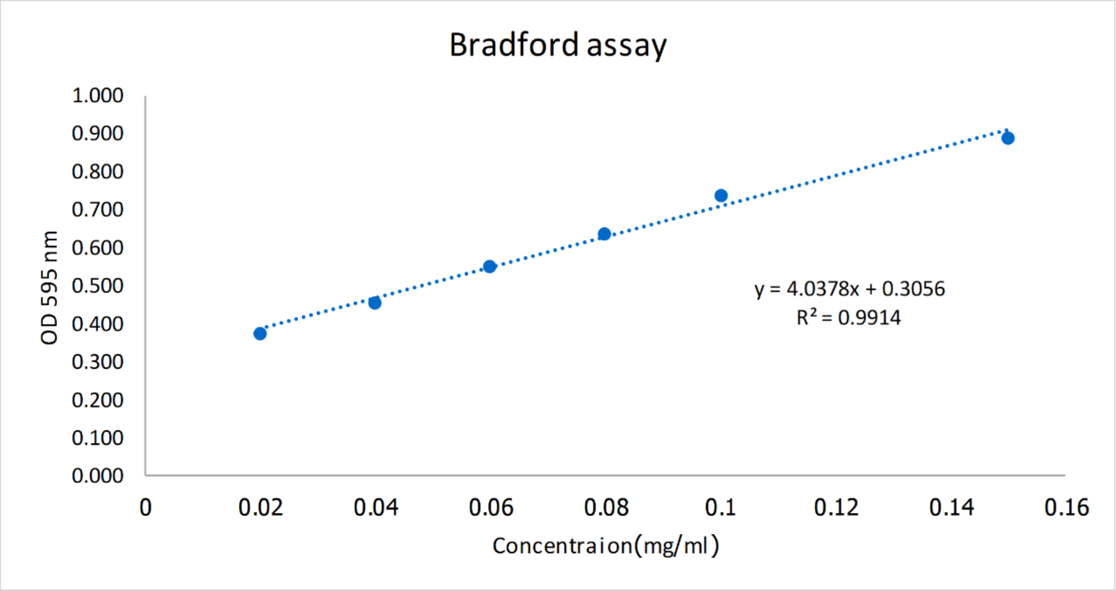

Figure S2. Standard curve of Bovine serum albumin (BSA) for protein content by Bradford assay.

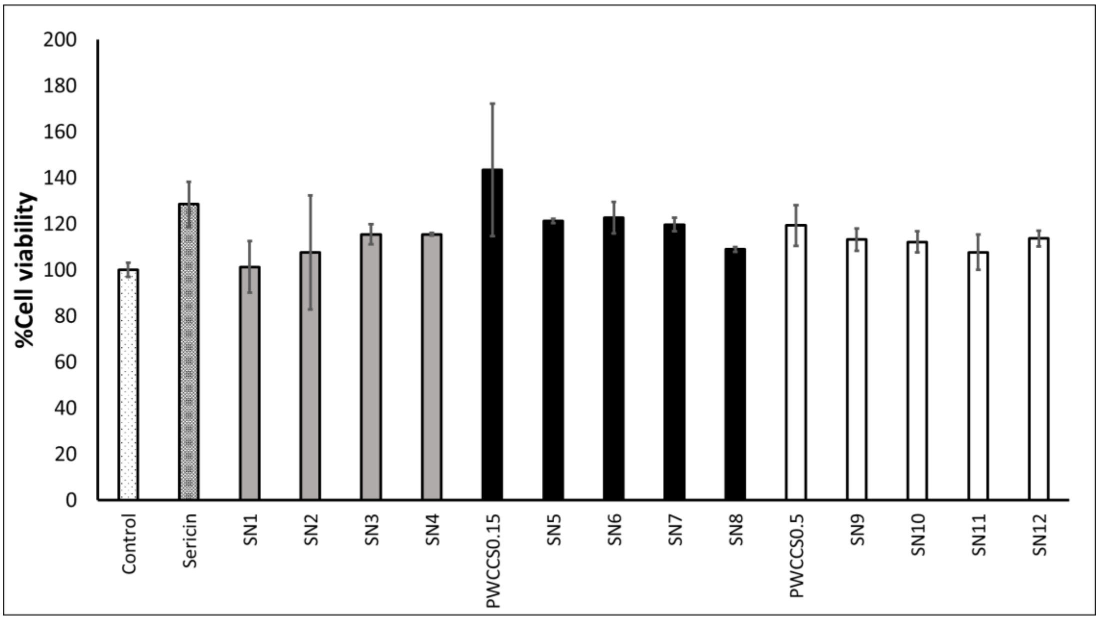

Figure S3. Effect of sericin, purple waxy corn cob extract solution 0.15% and 0.5% (PWCCS0.15 and PWCCS0.5), sericin-hydrogel formulations (SN1-SN12) on cell viability of RAW 264.7 cells. Data represent the mean ± SEM of three replicates.

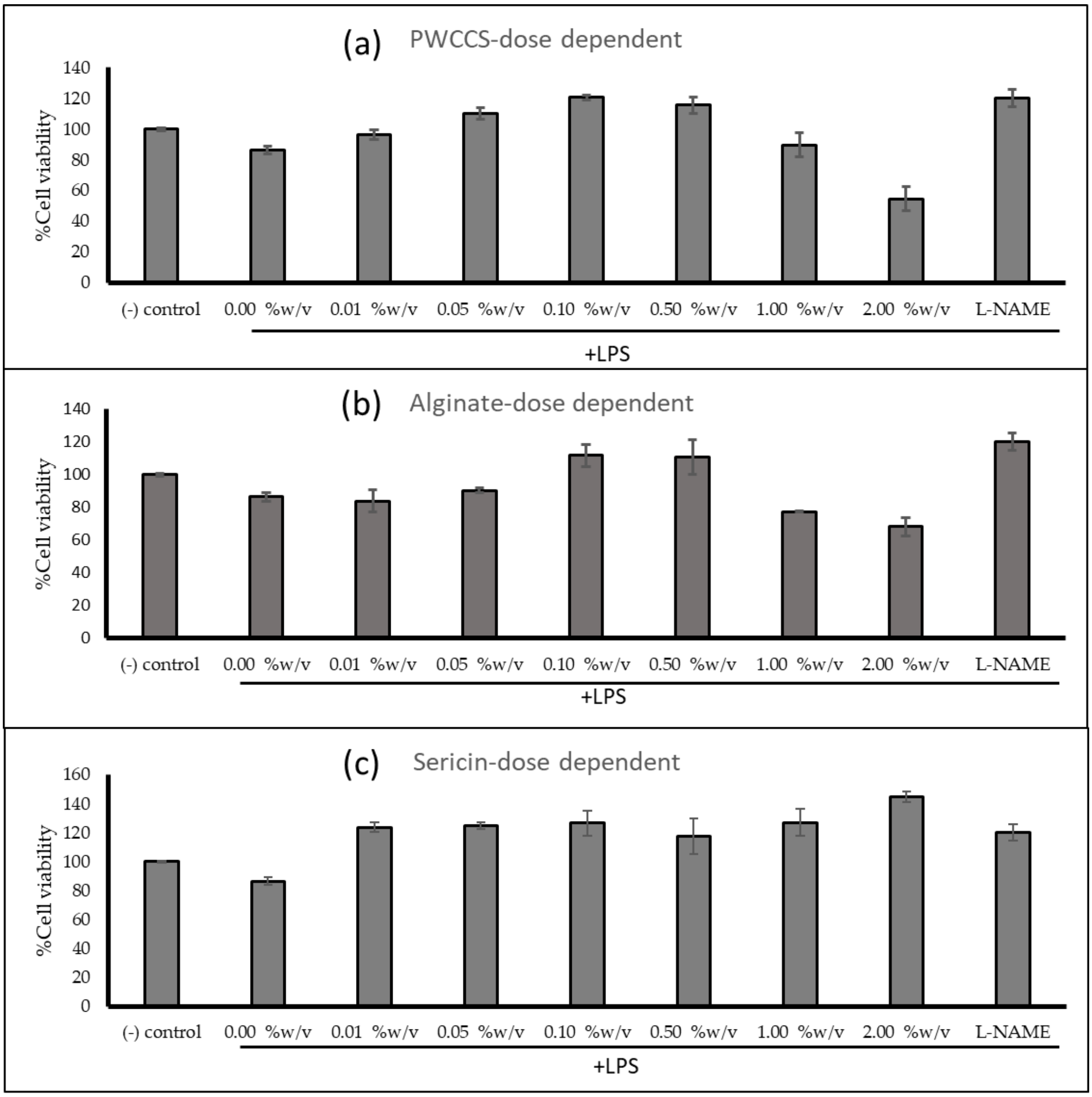

Figure S4. The %cell viability on LPS-stimulated RAW 264.7 cells for 0-2%w/v of PWCCS (a), alginate (b) sericin (c) and L-NAME 200 μM. Data represent the mean ± SEM of three replicates.

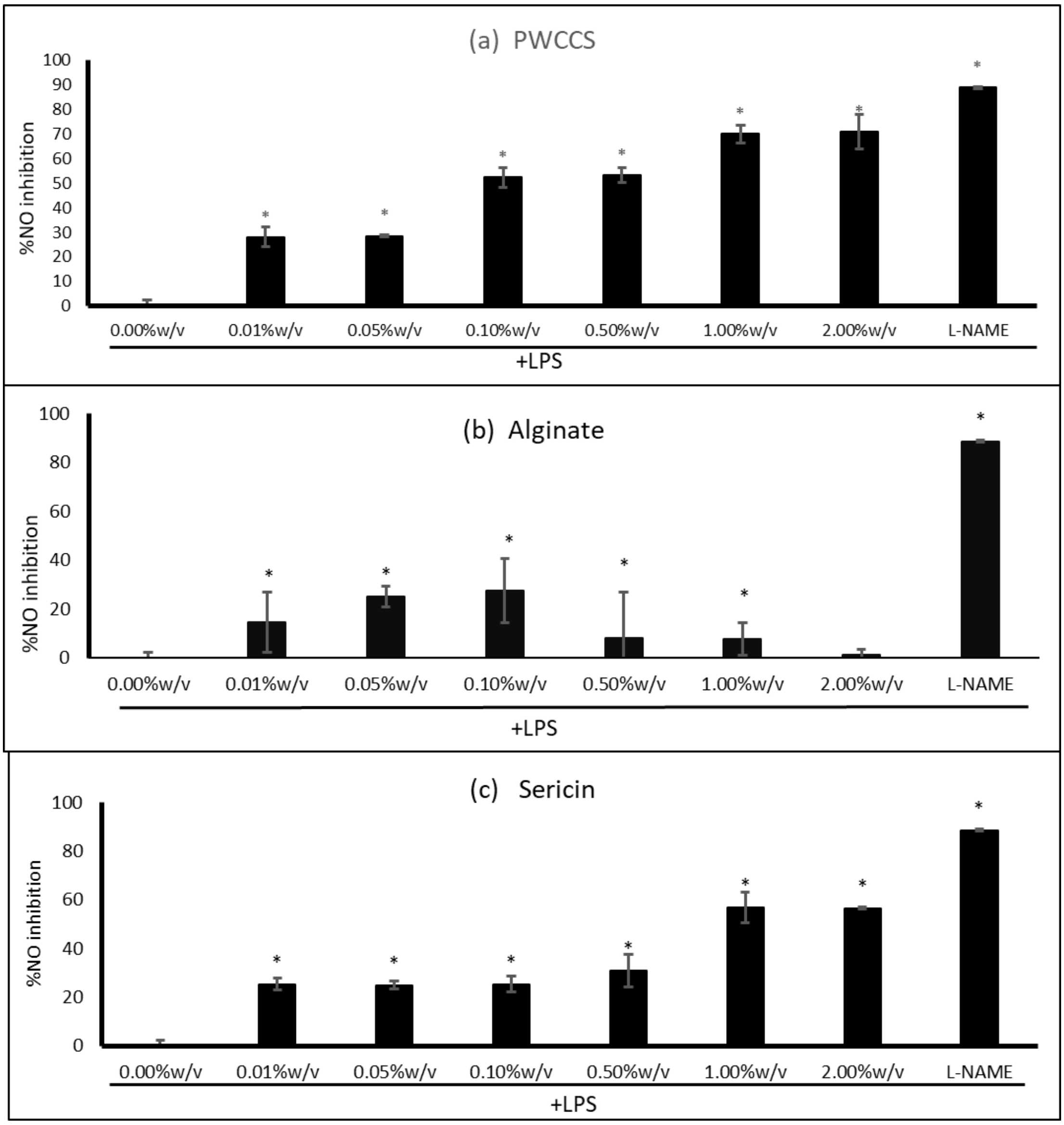

Figure S5. NO inhibition of extracts on LPS-stimulated RAW 264.7 cells (a) PWCCS (b) Alginate (c) Sericin and L-NAME 200 μM. Data represent the mean ± SEM of three replicates. (* $p < 0.05$, compared with compared with LPS treated cells (0%w/v).

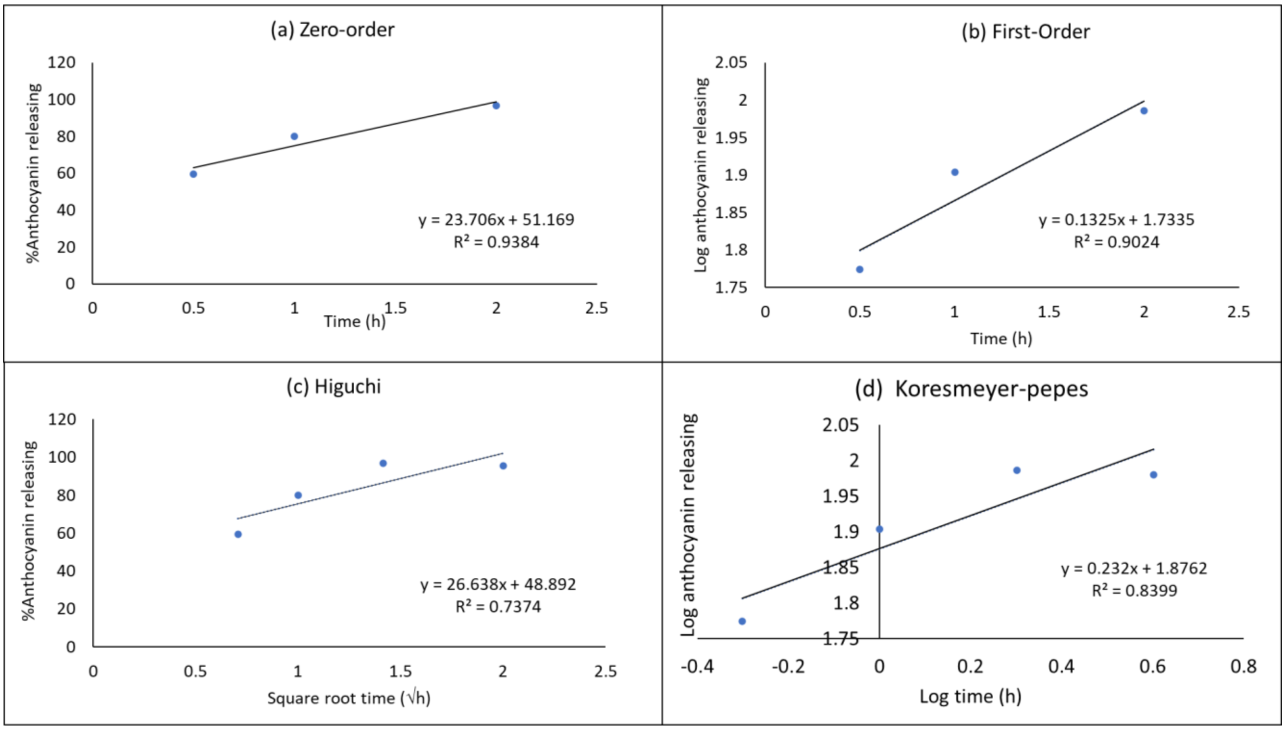

Figure S6. The kinetic model prediction of releasing for 0.15%PWCCS: (a) Zero order, (b) First order, (c) Higuchi and (d) Korsmeyer-Peppas.

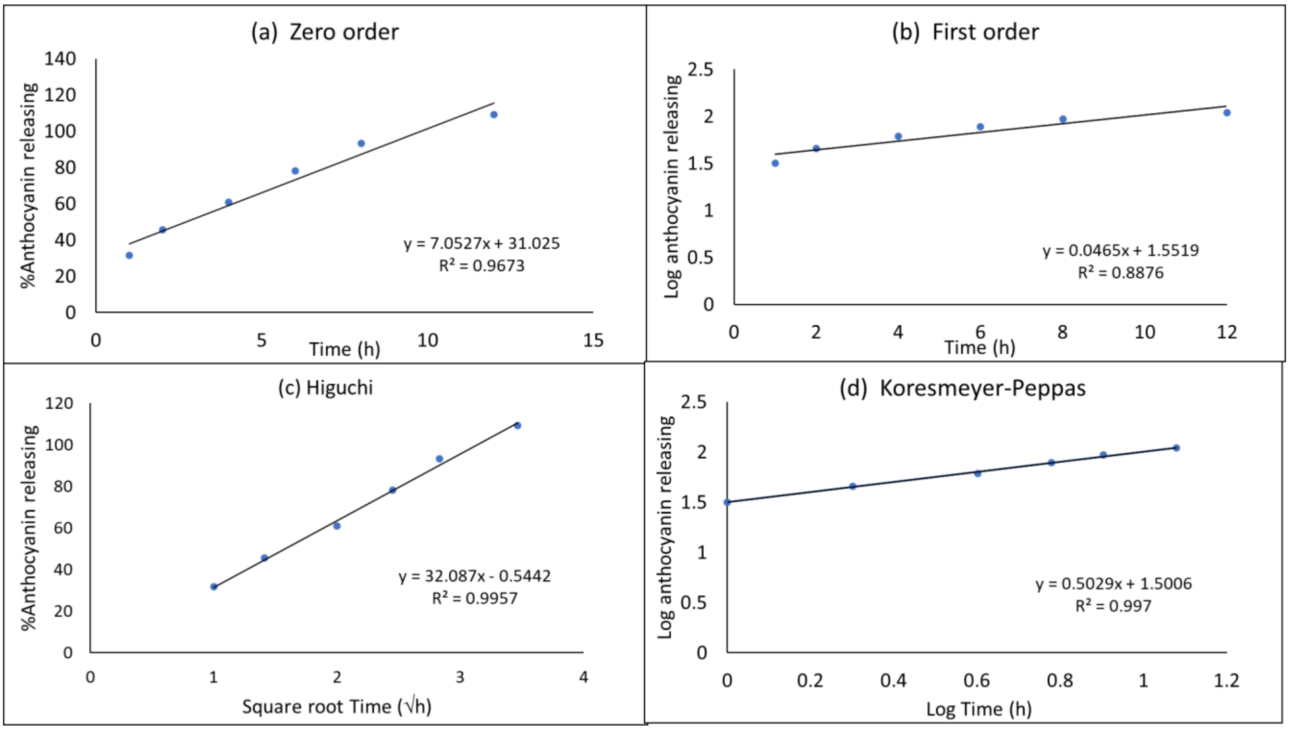

Figure S7. The kinetic model prediction of releasing for SN5 : (a) Zero order, (b) First order , (c) Higuchi and (d) Korsmeyer-Peppas.

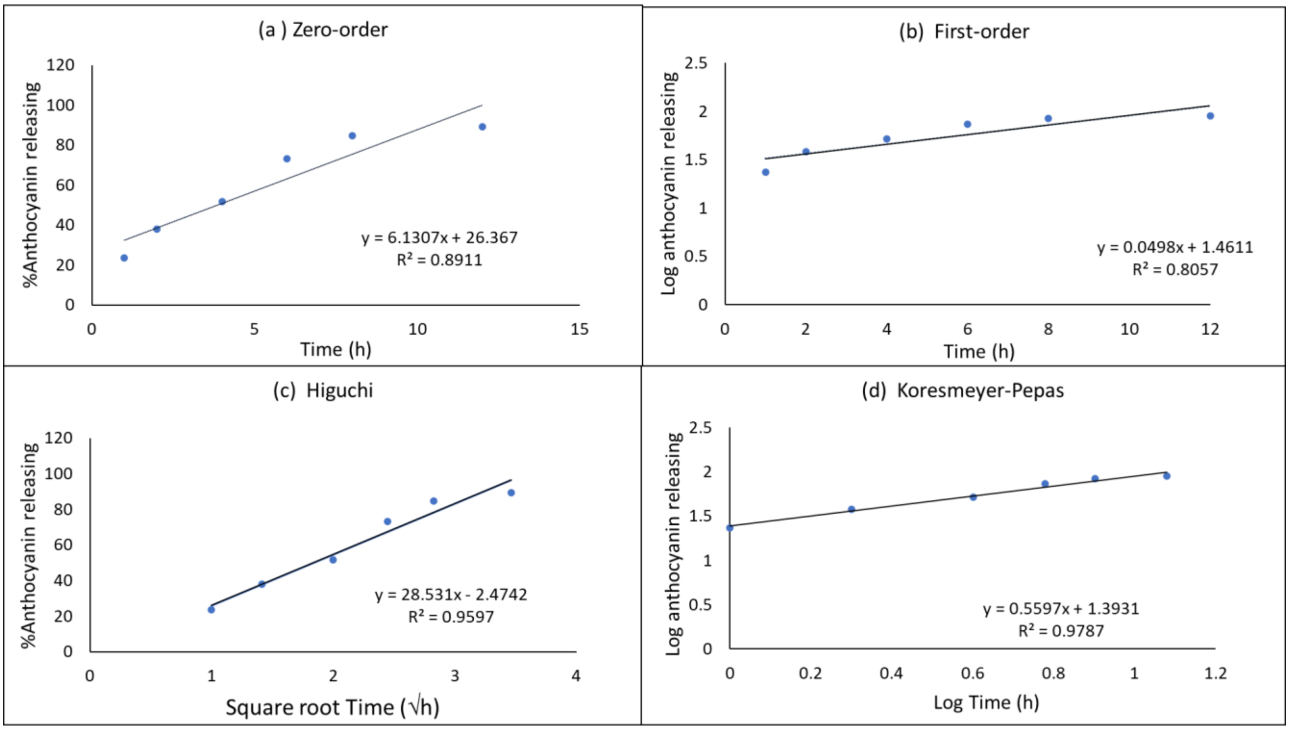

Figure S8. The kinetic model prediction of releasing for SN6: (a) Zero order, (b) First order , (c) Higuchi and (d) Korsmeyer-Peppas.

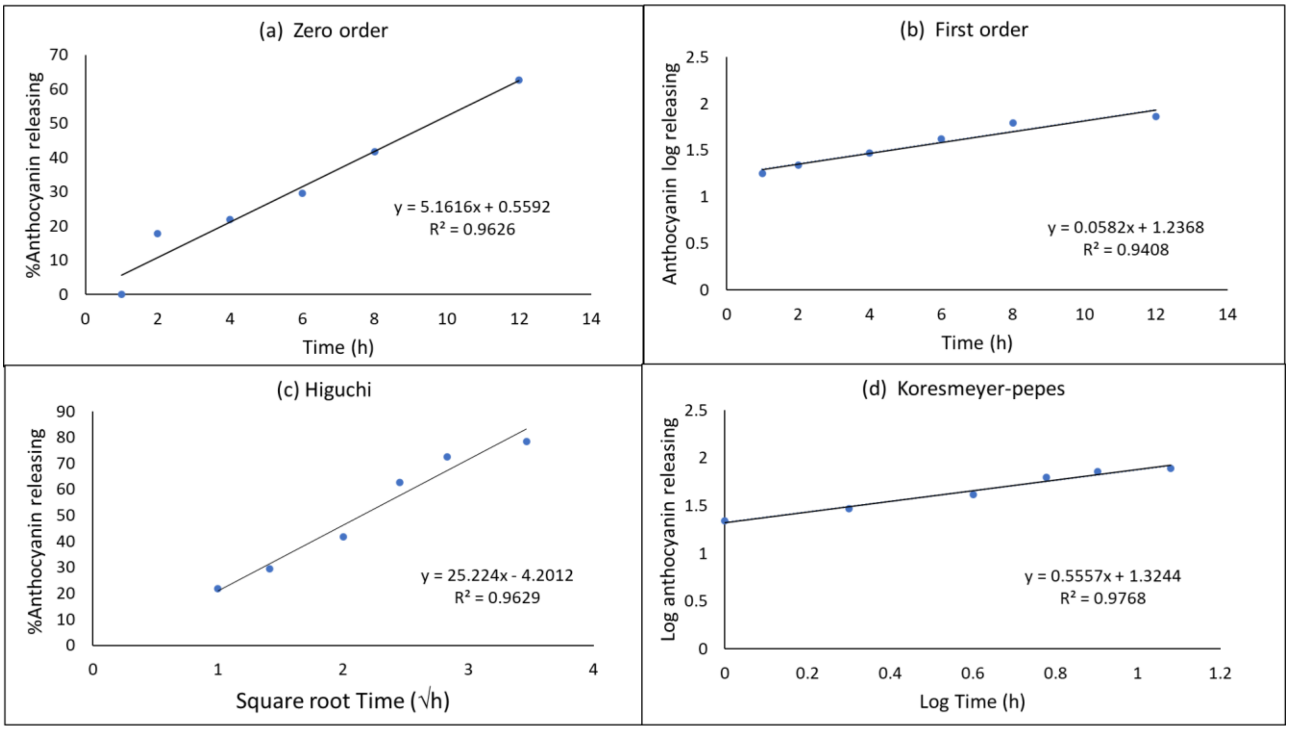

Figure S9. The kinetic model prediction of releasing for SN7: (a) Zero order, (b) First order , (c) Higuchi and (d) Korsmeyer-Peppas.

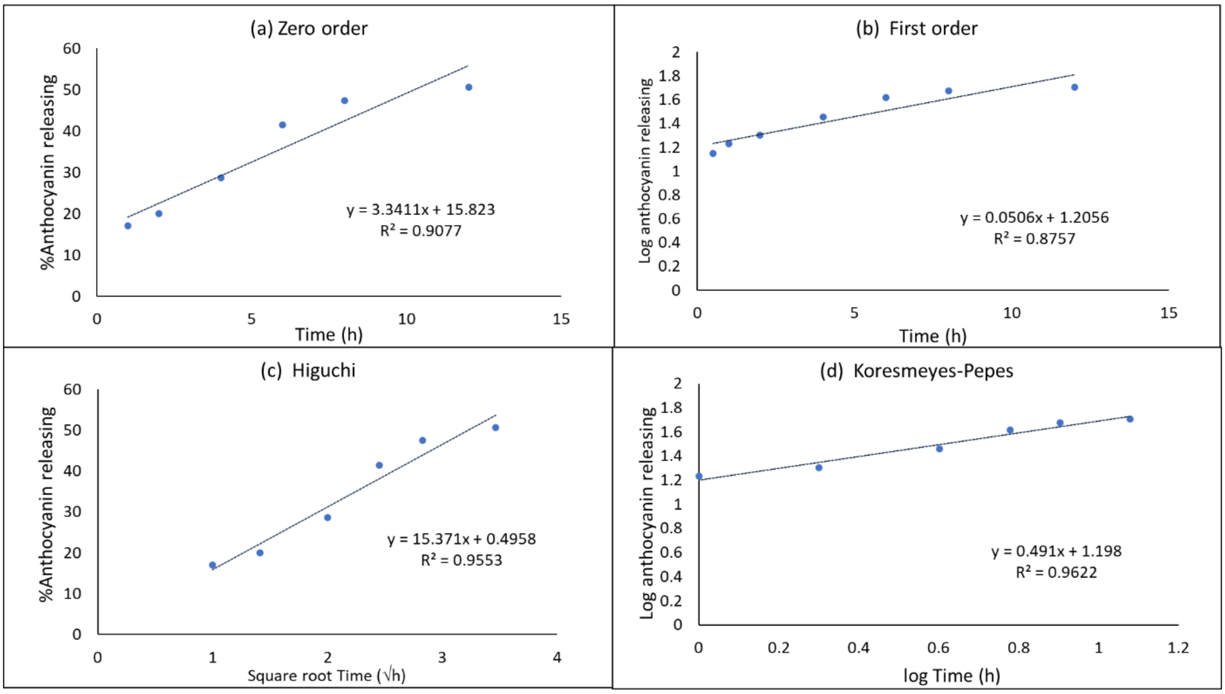

Figure S10. The kinetic model prediction of releasing for SN8: (a) Zero order, (b) First order, (c) Higuchi and (d) Korsmeyer-Peppas.

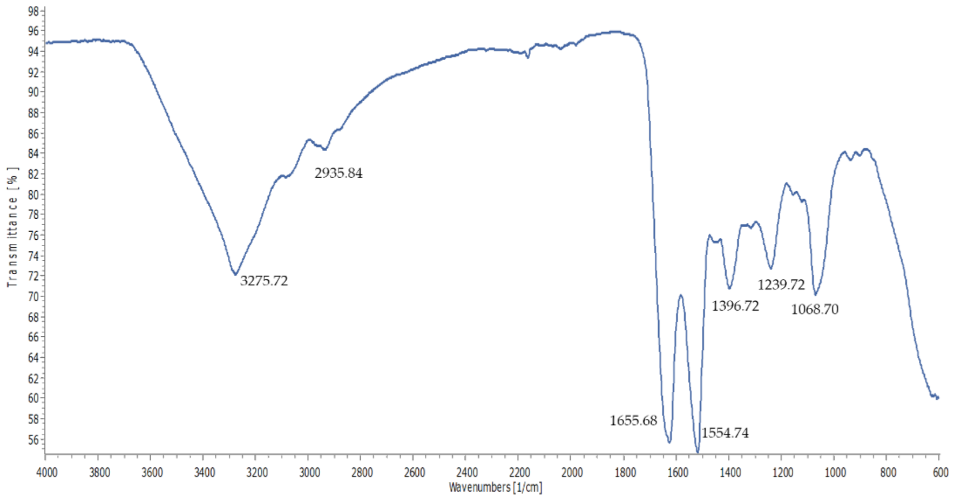

Sericin (cm^{-1})	Functional groups
3275.72	O–H stretch (overlap with NH-stretching)
2935.84	C H stretch (asymmetric)
1655.68	Amide I, C=O
1554.74	Amide II, N–H bending, C–N stretching
1396.72	C–H, OH bending (corresponding to OH groups of serine and threonine side chains)
1239.72	Amide III, C–N stretching, C=O bending
1068.70	C-OH stretching

Figure S11. IR spectrum of sericin

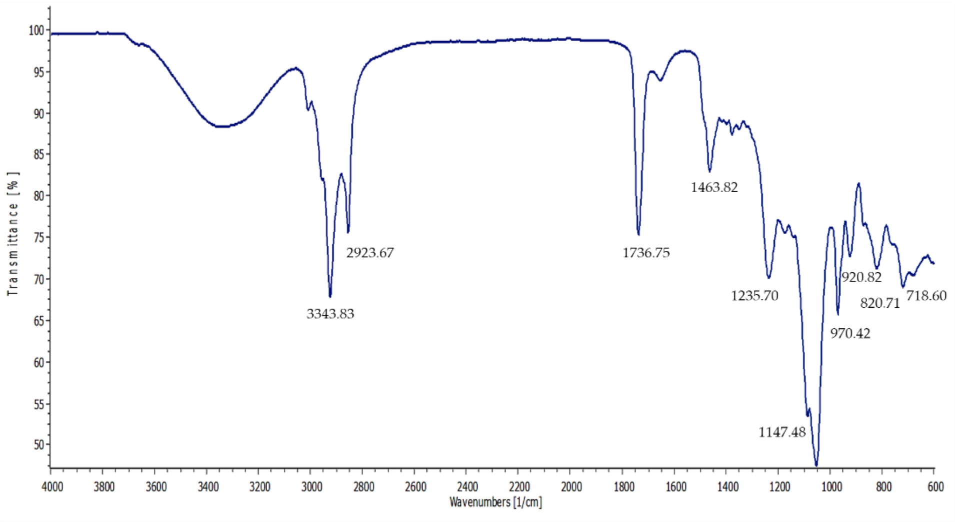

Purple corn cob extracts (cm^{-1})	Functional groups
3343.83	O–H stretch (phenolic OH)
2923.67	C–H stretch
1736.75	C=O stretch
1463.82	C–H bend
1235.70	C–O-stretch
1147.48	C–O–O stretch
970.42	C–H bending
920.82	C–H bending
820.71	C–H bending

Figure S12. IR spectrum of Purple waxy corn cob extracts (PWCC)

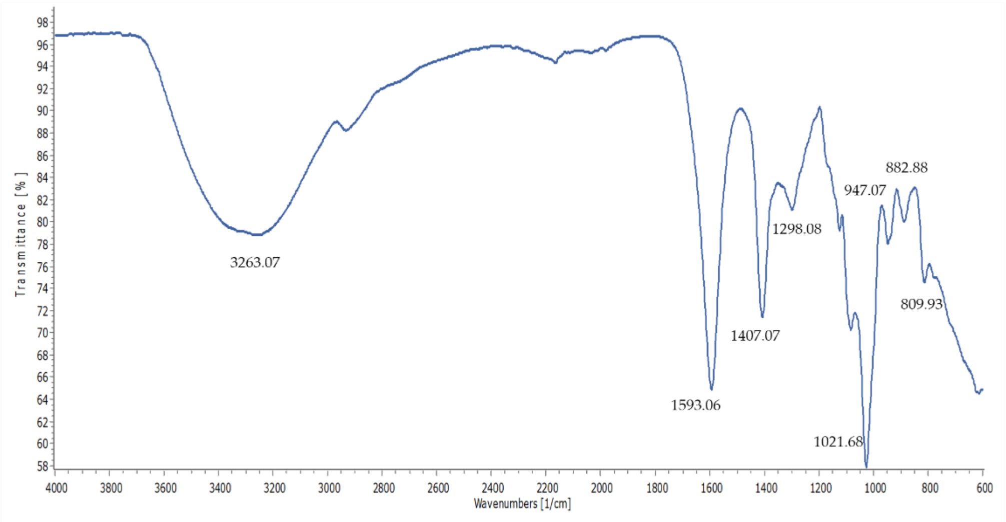

Alginate (cm^{-1})	Functional groups
3263.07	O–H stretch (polysaccharide)
1593.06	C–O–O stretch (asymmetric)
1407.07	C–O–O stretch(symmetric)
1298.08	C–H bending
1021.68	C–O stretch
947.08	C–H bending
882.88	C–H bending
809.93	C–H bending

Figure S13. IR spectrum of Alginate

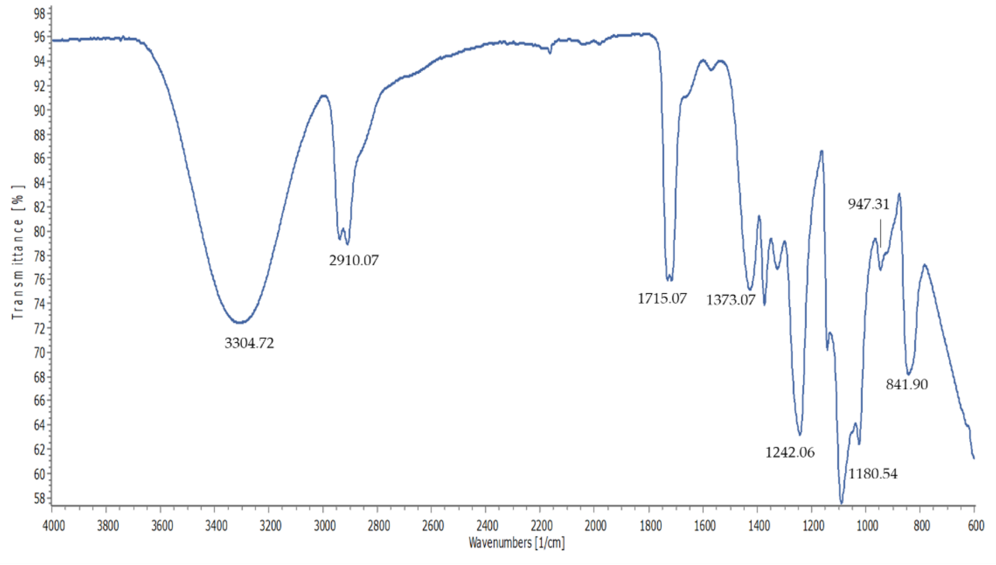

PVA (cm^{-1})	Functional groups
3304.72	O–H stretch
2910.07	C–H stretch of CH_2
1715.07	C=O stretch
1373.07	C–O stretching
1242.06	C–C stretch
1180.54	C–OH stretch
947.31	CH_2 rocking
841.90	C–C stretching

Figure S14. IR spectrum of PVA

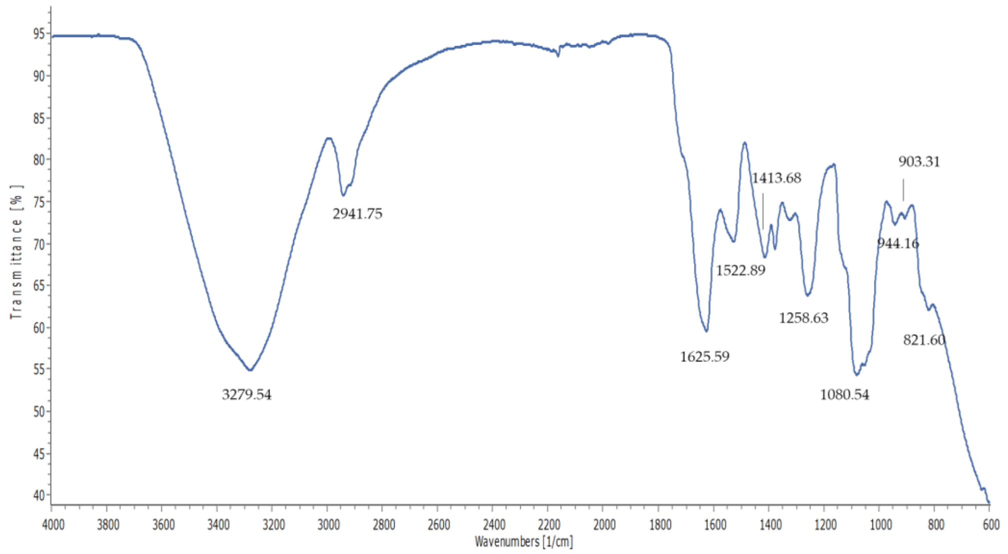

SN2 (cm^{-1})	Functional group
3279.54	O–H Stretch
2941.75	C–H Stretch
1625.59	Amide I, C=O (β-sheet structure))
1522.89	Amide II, N–H bending, C-N stretching
1413.68	C–H, O–H bending
1258.63	Amide III, C–N stretching, C=O bending
1080.54	C–OH stretching
944.16	C–H bending
903.31	C–H bending

Figure S15. IR spectrum of SN2

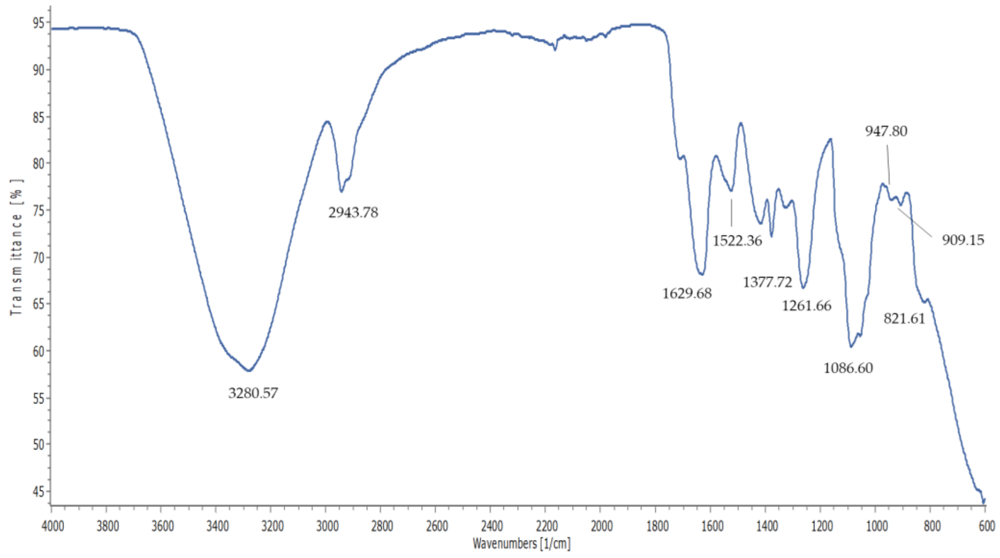

SN6 (cm^{-1})	Functional group
3280.57	O–H Stretch
2943.78	C–H Stretch
1629.68	Amide I, C=O (β-sheet structure))
1522.36	Amide II, N–H bending, C–N stretching
1377.72	C–H, OH bending
1261.66	Amide III, C–N stretching, C=O bending
1086.60	C–OH stretching
947.80	C–H bending
909.15	C–H bending
821.61	C–H bending

Figure S16. IR spectrum of SN6

Table S1. The protein content before and after storage condition.

**Sample**	**Storage at 4°C for 1 year**	
	**Before (mg/g)**	**After (mg/g)**
Sericin	29.28±0.50	29.05±0.13

Data represent the mean ± SEM of three replicates. The comparison in stability test was evaluated by Paired sample *t*-test. A p-value of less than 0.05 was considered statistically significant. Protein content of sericin extract before and after storage condition have no significant difference.
